# Alterations of Sphingolipid and Phospholipid Pathways and Ornithine Level in the Plasma as Biomarkers of Parkinson’s Disease

**DOI:** 10.3390/cells11030395

**Published:** 2022-01-24

**Authors:** Kuo-Hsuan Chang, Mei-Ling Cheng, Hsiang-Yu Tang, Cheng-Yu Huang, Hsiu-Chuan Wu, Chiung-Mei Chen

**Affiliations:** 1 Department of Neurology, Chang Gung Memorial Hospital Linkou Medical Center and College of Medicine, Chang Gung University, Taoyuan 333423, Taiwan; gophy5128@cgmh.org.tw (K.-H.C.); serenawu@cgmh.org.tw (H.-C.W.); 2 Department of Biomedical Sciences, Chang Gung University, Taoyuan 33323, Taiwan; chengm@mail.cgu.edu.tw; 3 Metabolomics Core Laboratory, Healthy Aging Research Center, Chang Gung University, Taoyuan 333323, Taiwan; tangshyu@gmail.com (H.-Y.T.); chenyu7015@gmail.com (C.-Y.H.); 4 Clinical Metabolomics Core Laboratory, Chang Gung Memorial Hospital, Taoyuan 333426, Taiwan

**Keywords:** Parkinson’s disease, biomarker, metabolomics, sphingomyelin, phosphatidylethanolamine, phosphatidylcholine

## Abstract

The biomarkers of Parkinson’s disease (PD) remain to be investigated. This work aimed to identify blood biomarkers for PD using targeted metabolomics analysis. We quantified the plasma levels of 255 metabolites in 92 PD patients and 60 healthy controls (HC). PD patients were sub-grouped into early (Hoehn–Yahr stage ≤ 2, n = 72) and advanced (Hoehn–Yahr stage > 2, n = 20) stages. Fifty-nine phospholipids, 3 fatty acids, 3 amino acids, and 7 biogenic amines, demonstrated significant alterations in PD patients. Six of them, dihydro sphingomyelin (SM) 24:0, 22:0, 20:0, phosphatidylethanolamine-plasmalogen (PEp) 38:6, and phosphatidylcholine 38:5 and 36:6, demonstrated lowest levels in PD patients in the advanced stage, followed by those in the early stage and HC. By contrast, the level of ornithine was highest in PD patients at the advanced stage, followed by those at the early stage and HC. These biomarker candidates demonstrated significant correlations with scores of motor disability, cognitive dysfunction, depression, and quality of daily life. The support vector machine algorithm using α-synuclein, dihydro SM 24:0, and PEp 38:6 demonstrated good ability to separate PD from HC (AUC: 0.820). This metabolomic analysis demonstrates new plasma biomarker candidates for PD and supports their role in participating PD pathogenesis and monitoring disease progression.

## 1. Introduction

Parkinson’s disease (PD) is an age-related neurodegenerative disease characterized by bradykinesia, limb rigidity, postural instability, and freezing of gait. The pathology is associated with progressive degeneration of dopaminergic neurons in the substantia nigra [1]. The pathogenesis of neurodegeneration in PD has not been fully disclosed. Several pathogenic pathways have been identified, including accumulation of aberrant or misfolded proteins, mitochondrial and metabolic dysfunction, increased oxidative stress and inflammation, and failure of mitophagy and autophagolysosome [1]. Currently, effective treatments to prevent disease progression or modify the disease course for PD are not available. The main hurdle in developing an effective treatment for PD is the lack of objective and useful biomarkers to indicate early disease progression and to test the efficacy of potential treatments. The establishment of PD-specific molecular biomarkers, particularly in blood, could be helpful in revealing new steps of pathogenesis, detecting the disease at early stages, indicating disease severity, and monitoring the therapeutic effect of potential disease modifiers [2].

Metabolites are compartment-specific in the sense that these substances are localized in different body fluids, cells, and tissues that may participate in dissimilar biochemical reactions. A metabolite may be produced by catabolic reactions in a tissue, while it is utilized for anabolism in another tissue. It follows that any changes in metabolites in different body fluids, cells, and tissues may have a different interpretation and be of different relevance to physiology and pathophysiology. In PD, metabolic profiles revealed by various metabolomics platforms showed disturbance of a number of PD-associated metabolic pathways, including catecholamine, amine, and polyamine metabolism [3,4,5,6], caffeine, xanthine, hypoxanthine, kynurenine and, purine pathways [4,7,8], amino acid [4,9], and fatty acids [9], as well as redox markers [4,10]. The redox markers include increased 8-hydroxy-2-deoxyguanosine [10], glutathione [10], and biliverdine [4], as well as reduced uric acid [10], bilirubin, and ergothioneine [4] levels in the plasma of PD patients. Herein we measured plasma levels of 255 metabolites in PD patients to identify candidate metabolic biomarker(s) and pathomechanistic pathway(s) of PD by using liquid chromatography mass spectrometer (LC-MS) metabolomics analysis. The identified biomarker candidates were further correlated with clinical parameters for movement disability, cognitive dysfunction, and depression, as well as implemented to machine learning model to establish an algorithm for diagnosing PD.

## 2. Materials and Methods

### 2.1. Patient Recruitment

Patients with PD were recruited from the neurology clinics of Chang Gung Memorial Hospital. The diagnosis of PD was based on the UK PD Society Brain Bank clinical diagnostic criteria by two neurologists specialized in movement disorders (KH Chang and CM Chen) [11]. Demographic information, levodopa equivalent daily dose (LEDD) [12], Unified Parkinson’s Disease Rating Scale (UPDRS) [13], and Hoehn and Yahr (H&Y) stage [14] were recorded for each patient. All patients underwent a battery of neuropsychological assessments, including the Mini-Mental State Examination (MMSE) [15], Montreal Cognitive Assessment (MoCA) [16], Clinical Dementia Rating (CDR) [17], Beck Depression Inventory II (BDI-II) [18], Hamilton Depression Rating Scale (HAM-D) [19], Activities of Daily Living (ADL) [20], the Parkinson’s Disease Questionnaire (PDQ-39) [21], and Neuropsychiatric Inventory Questionnaire (NPI) [22]. PD patients with H&Y stage 1–2 were defined as at the early stage, while those with H&Y stage higher than 2 were classified as at the advanced stage. Sex- and age-matched healthy controls (HC) were randomly recruited from neurology outpatient clinics. All subjects were Taiwanese and primarily of East Asian descent. All subjects had no systemic infection, chronic renal failure, cardiac or liver dysfunction, malignancies, autoimmune diseases, stroke, or neurodegenerative diseases other than PD. Blood samples for metabolomics analysis were collected from subjects who were asked to be on fasting overnight for 12 h. 

### 2.2. Amino Acid Analysis

Amino acid analysis was performed as previously described [23]. Briefly, the 100 μL of plasma samples were precipitated by 10% sulfosalicylic acid. The derivatization was initiated by the addition of 20 μL of 6-aminoquinolyl-N-hydroxysuccinimidyl carbamate in acetonitrile and analyzed by an ultra-performance liquid chromatography System. The derived amino acids were detected using a PDA detector at a wavelength of 280 nm. 

### 2.3. Biogenic Amines Analysis with Liquid Chromatography Mass Spectrometry

Sample were derived as previous description [23] and analyzed with a manufacturer’s instruction of Kairos Amino Acid Kit (Waters Corp., Milford, MA, USA). Briefly, the chromatographic separation was achieved on a CORTECS C18 reversed-phase column (2.1 × 150-mm i.d., 1.6 μm, Waters Corp., Milford, MA, USA) at 55 °C with mobile phase A (0.1% formic acid in water, *v*/*v*) and mobile phase B (0.1% formic acid in acetonitrile, *v*/*v*) and the flow rate was set at 0.5 mL/min. The parameters of MSMS were as follows: capillary voltage 2 kV; desolvation gas flow 1000 L/h; desolvation temperature 500 °C; source temperature 150 °C. 

### 2.4. Tryptophan Metabolites Analysis with Liquid Chromatography Mass Spectrometry 

Tryptophan analysis was performed as previously described [8]. Briefly, the 20 μL plasma sample was extracted with methanol containing internal standards and analyzed in an LC-MS/MS system (Waters Corp., Milford, MA, USA). System operation and data acquisition were controlled using Mass Lynx software, and targeted metabolic data were analyzed by TargetLynx (Waters Corp., Milford, MA, USA).

### 2.5. Acylcarnitines Analysis with Liquid Chromatography Mass Spectrometry 

The 20 μL plasma sample was extracted with 180 μL methanol containing internal standards in a 96-well plate with a filter membrane. After centrifugation, 50 μL of the filtrate was transferred to a microtiter plate and diluted 4 times for analysis [24]. The chromatographic separation was achieved on a C8 column (2.1 × 100 mm i.d., 1.7 μm, Waters Corp., Milford, MA, USA) at 45 °C with mobile phase A (0.1% formic acid in water, *v*/*v*) and mobile phase B (acetonitrile), and the flow rate was set at 0.5 mL/min. The gradient profile was as follows: linear gradient 0–2.5% B, 0.5 min, 2.5–12% B, 2 min, 12–36% B, 0.5 min, and keep 36% B, 1 min, 36–100% B, 0.2 min, and keep 100% B, 1.3 min. The column was then re-equilibrated for 1.4 min. The parameters of MS were as follows: capillary voltage 1 kV; desolvation gas flow 1000 L/h; desolvation temperature 550 °C; source temperature 150 °C. Acylcarnitines were quantified in positive electrospray ionization mode.

### 2.6. Phospholipid Analysis with Liquid Chromatography Mass Spectrometry 

The 490 μL of pre-cooled isopropanol containing isotope-labeled standard and 10 μL of plasma were added to a 96-well plate with filter membrane. After centrifugation, the filtrate was diluted with an equal volume of 50% acetonitrile for free fatty acids analysis. Sequentially, the diluted sample was diluted again 10 times for phospholipid analysis. 

For phospholipid separation, a C18 (2.1 mm × 100 mm, 1.7 µm, Waters Corp., Milford, MA, USA) column was performed for chromatographic separation at 60 °C with mobile phase A (acetonitrile/water (4:6) with 10 mM ammonium formate) and mobile phaser B (isopropanol/acetonitrile (90:10) with 10 mM ammonium formate) at flow rate 0.45 mL/min. The initial LC gradient conditions were 40% buffer B, increasing to 99% B within 10 min, then back to 40% B for 0.1 min and kept for 2 min for re-equilibration. The desolvation gas was set at 1000 L/h at a temperature of 500 °C; the cone gas was set at 150 L/h, and the source temperature was set at 150 °C. The capillary voltage and cone voltage were set to 1000 and 25 V, respectively. The semi-quantification of phospholipid was determined by comparing the abundance with the isotope-labeled lipid standard. 

Free fatty acids analysis was followed the manufacturer’s application note with modification (72000627en, Waters Corp., Milford, MA, USA). Briefly, a CORTECS T3 (2.1 mm × 30 mm, 2.7 µm, Waters Corp., Milford, MA, USA) column was set at 60 °C and the flow rate was set at 0.65 mL/min with 0.01% formic acid, and isopropanol/acetonitrile (50:50) with 0.01% formic acid. The desolvation gas was set at 1200 L/h at a temperature of 600 °C; the cone gas was set at 150 L/h and the source temperature was set at 150 °C. The capillary voltage and cone voltage were set to 2000 and 45 V, respectively. The quantification of fatty acids was determined by comparing the abundance with the sample of known concentration. 

### 2.7. Measurement of α-Synuclein in Plasma

We used an immunomagnetic reduction assay to measure the plasma levels of total α-synuclein, as previously described [25]. 

### 2.8. Statistical Analysis

Continuous variables were presented as mean and standard deviation (SD) and analyzed by Student’s *t*-test or one-way ANOVA with false discovery rate (FDR) adjustment to correct multiple tests where appropriate. Categorical variables were presented as counts and percentages and analyzed by chi-square test. The clinical variables and metabolites were analyzed using orthogonal partial least squares discriminant analysis (OPLS-DA) through the web-based metabolomics software MetaboAnalyst 5.0. The variable importance (VIP) in the projection of each metabolite in the model was calculated to indicate its contribution to the classification. A higher VIP value indicates a stronger contribution to discrimination between groups. VIP values greater than 1.0 were considered significantly different. Pearson correlation was applied to evaluate the relationship between the levels of metabolites and clinical parameters. An analysis of the receiver operating characteristic (ROC) curve was used to measure the ability of individual molecules to distinguish PD patients from HCs. Selected molecules were further introduced to the support vector machine (SMV) algorithm. The models’ performance estimation was further analyzed by ROC curves generated by Monte-Carlo cross-validation using balanced sub-sampling. Two-thirds of subjects were used to build classification models, which was validated on the 1/3 of subjects that were left out. To produce a smooth ROC curve, 100 cross validations were performed, and the results were averaged to generate the plot.

## 3. Results

### 3.1. Demography and Clinical Presentations of Subjects

A total of 92 PD patients (71 patients at early stage and 21 patients at advanced stage) and 60 sex- and age-matched HC were recruited in this study (Table 1). Patients with PD demonstrated significantly higher scores in CDR, BDI-II, HAM-D, PDQ-39, and NPI, compared with HC (all *p* < 0.001). The scores of MMSE, MoCA and ADL were significantly lower in PD patients compared with HC (*p* = 0.005~<0.001). Not surprisingly, PD patients at the advanced stage had older age, higher scores of UPDRS and H&Y stage, and higher LEDD compared with those at the early stage (*p* = 0.002~<0.001). The scores of CDR, BDI-II, HAM-D, PDQ-39, NPI were significantly higher in PD patients at the advanced stage compared with those at the early stage (all *p* < 0.001). PD patients at the advanced stage displayed lower scores of MoCA and ADL compared with those at the early stage (all *p* < 0.001). Although the number of diabetic patients in PD group (12 patients, 13.04%) was larger than HC group (3 patients, 5%), the levels of pre-prandial glucose, triglyceride, and cholesterol and body mass index were similar among different groups.

### 3.2. Targeted Metabolomics Analysis

**Figure 1:**
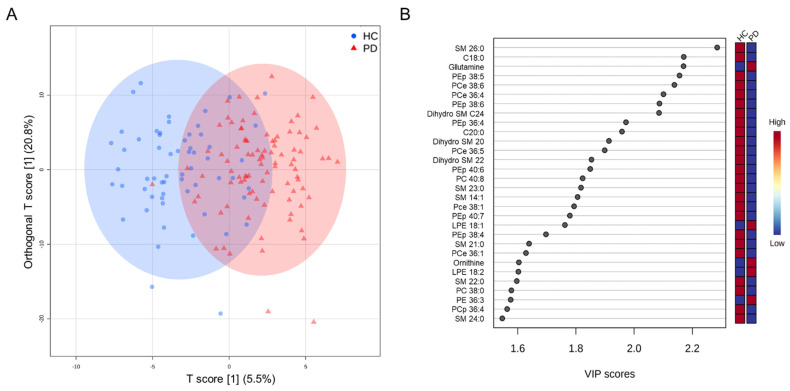
Orthogonal partial least squares-discriminant analysis (OPLS-DA) analysis between the healthy controls (HC, n = 60) and patients with Parkinson’s disease (PD) group (n = 92). (**A**) Orthogonal partial least squares discriminant analysis (OPLS-DA) demonstrates a separation of metabolites between two groups (R^2^Y = 0.22, Q^2^ = 0.15). R^2^Y, cumulative variation in the Y matrix; Q^2^, predictive performance of the model. (**B**) Top 30 metabolites with variable importance in the projection (VIP) score > 1.0 indicating their contribution to the classification in the OPLS-DA model. C: ceremide; LPE: lysophosphatidylethanolamine; PC: phosphatidylcholine; PCe: phosphatidylcholine-ether; PCp: phosphatidylcholine- plasmalogen; PEp: phosphatidylethanolamine-plasmalogen; SM: sphinogomyelin.

Plasma concentrations of 255 metabolites, including 170 phospholipids, 24 free fatty acids, 22 amino acids, 16 biogenic amines, 15 acylcarnitines, and 8 tryptophan metabolites, were quantitated. The OPLS-DA for all metabolites could separate PD from HC (R2Y, 0.22; Q2, 0.15, Figure 1A), while 90 metabolites had VIP score > 1.0 (Figure 1B). There were 72 metabolites, including 13 sphingomyelins (SMs), 5 dihydro sphingomyelins (dihydro SMs), 33 phosphatidylcholines (PCs), 8 phosphatidylethanolamines (PEs), 3 free fatty acids, 10 ceramides, and biogenic amines/amino acids, demonstrating significantly different plasma levels between PD and HC (Table 2 and Supplementary Table S1).

**Figure 2:**
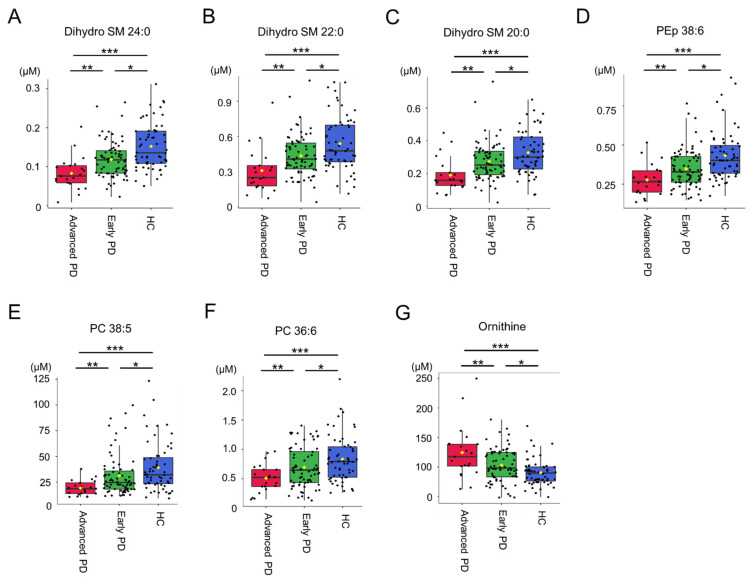
Differences in (**A**) dihydro sphingomyelin (SM) 24:0, (**B**) dihydro SM 22:0, (**C**) dihydro SM 22:0, (**D**) phosphatidylethanolamine-plasmalogen (PEp) 38:6, (**E**) phosphatidylcholine (PC) 38:5, (**F**) PC 36:6, and (**G**) ornithine among Parkinson’s disease (PD) patients at early (early PD) and advanced stages (advanced PD), and the healthy controls (HC). *: Statistically significant between early and advanced PD. *p* < 0.05, One-way analysis of variance with Bonferroni correction. **: Statistically significant between early PD and HC. *p* < 0.05, One-way analysis of variance with Bonferroni correction. ***: Statistically significant between advanced PD and HC. *p* < 0.05. One-way analysis of variance with Bonferroni correction.

Only 6 metabolites, including dihydro SM 24:0 (advanced vs. early vs. HC: 0.08 ± 0.04 μM vs. 0.12 ± 0.05 μM vs. 0.152 ± 0.059, advanced vs. early *p* = 0.003, advanced vs. HC *p* < 0.001, early vs. HC *p* < 0.001, Figure 2A), dihydro SM 22:0 (advanced vs. early vs. HC: 0.31 ± 0.19 μM vs. 0.44 ± 0.18 μM vs. 0.539 ± 0.233, advanced vs. early *p* = 0.006, advanced vs. HC *p* < 0.001, early vs. HC *p* = 0.005, Figure 2B), dihydro SM 20:0 (advanced vs. early vs. HC: 0.19 ± 0.10 μM vs. 0.27 ± 0.12 μM vs. 0.327 ± 0.133, advanced vs. early *p* = 0.004, advanced vs. HC *p* < 0.001, early vs. HC *p* = 0.017, Figure 2C), phosphatidylethanolamine-plasmalogen (PEp) 38:6 (advanced vs. early vs. HC: 0.28 ± 0.10 μM vs. 0.35 ± 0.12 μM vs. 0.433 ± 0.158, advanced vs. early *p* = 0.019, advanced vs. HC *p* < 0.001, early vs. HC *p* = 0.001, Figure 2D), phosphatidylcholine (PC) 38:5 (advanced vs. early vs. HC: 19.37 ± 7.40 μM vs. 31.33 ± 20.01 μM vs. 39.095 ± 23.968, advanced vs. early *p* = 0.009, advanced vs. HC *p* < 0.001, early vs. HC *p* = 0.045, Figure 2E) and PC 36:6 (advanced vs. early vs. HC: 0.84 ± 0.40 μM vs. 0.70 ± 0.34 μM vs. 0.51 ± 0.24, advanced vs. early *p* = 0.022, advanced vs. HC *p* = 0.001, early vs. HC *p* = 0.032, Figure 2F) demonstrated significant differences among HC, and early and advanced, with PD patients at advanced stage showing the lowest, followed by those at early stage, and HC. Ornithine showed the highest level in plasma of PD patients at advanced stage (124.23 ± 45.04 μM), followed by those at early stage (102.65 ± 28.80, advanced vs. early *p* = 0.010) and HC (90.217 ± 22.245, advanced vs. HC < 0.001, early vs. HC *p* = 0.007, Figure 2G). These 7 metabolites were selected as biomarker candidates for further correlation with clinical parameters in PD group.

### 3.3. Clustering and Correlation Analysis

**Figure 3:**
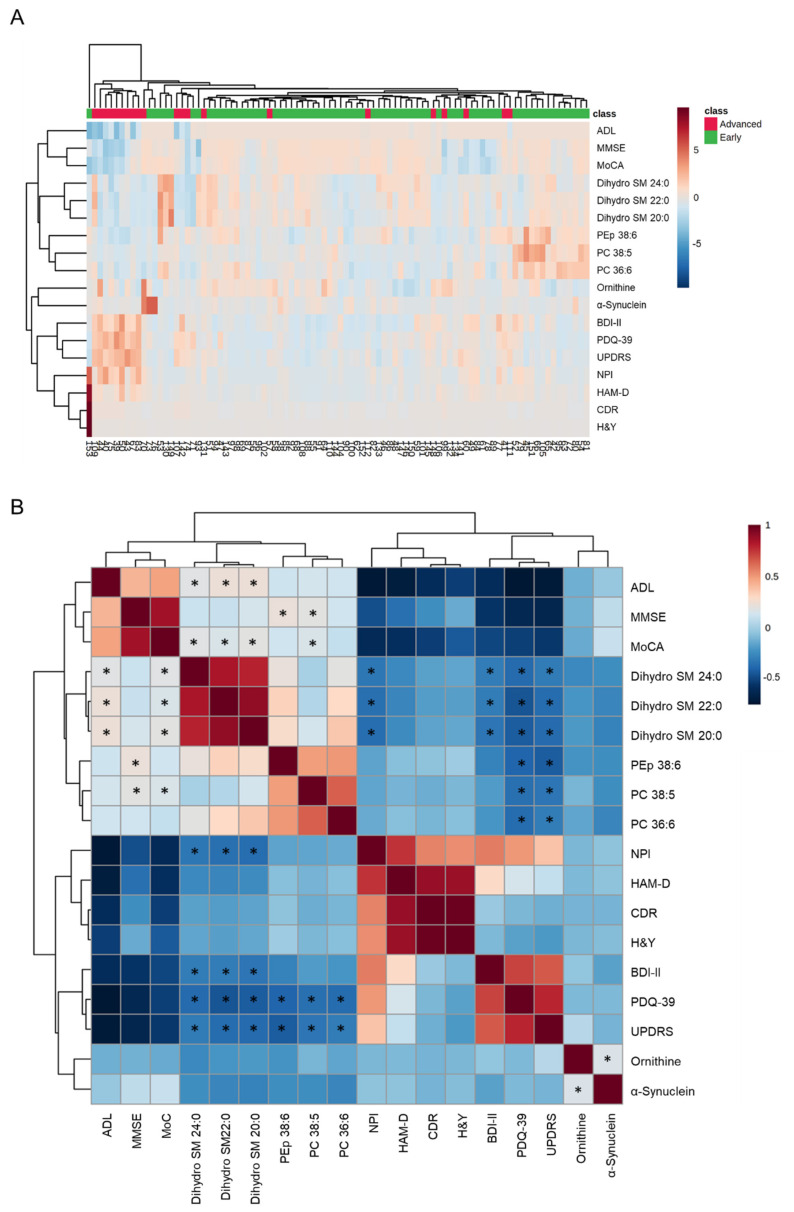
The correlations between identified metabolites and clinical parameters. (**A**) Heatmap of the hierarchical clustering. The dendrogram on top shows the clustering of patients, and the dendrogram on the side shows the clustering of features. The colors on top of the heatmap represent Parkinson’s disease patients at the early or advanced stage. The colors in the heatmap represent normalized intensities, scaled to mean of zero and unit variance for each feature. (**B**) Correlation matrix for clinical parameters and metabolites. Negative correlations are indicated with blue and positive correlations are indicated with red. *: Statistically significant correlations between clinical parameters and plasma levels of metabolites, *p* < 0.05, Pearson correlation. ADL: activities of daily living; BDI-II: Beck Depression Inventory II; CDR: Clinical Dementia Rating; HAM-D: Hamilton Depression Rating Scale; LEDD: Levodopa Equivalent Daily Dose; MMSE: Mini-Mental State Examination; MoCA: Montreal Cognitive Assessment; NPI: Neuropsychiatric Inventory Questionnaire; PDQ-39: Parkinson’s Disease Questionnaire; PC: phosphatidylcholine; PEp: phosphatidylethanolamine-plasmalogen; SM: sphingomyelin; UPDRS: Unified Parkinson’s Disease Rating Scale.

The hierarchical clustering heatmaps and correlation matrix using the selected biomarker candidates and clinical parameters of PD patients were demonstrated in Figure 3. Most of PD patients at advanced stage were aggregated in the same cluster (Figure 3A). The scores for UPDRS were negatively correlated with plasma levels of dihydro SM 24:0 (r = −0.227, *p* = 0.029), dihydro SM 22:0 (r = −0.276, *p* = 0.008), dihydro SM 20:0 (r = −0.285, *p* = 0.006), PEp 38:6 (r = −0.325, *p* = 0.002), PC 38:5 (r = −0.243, *p* = 0.020) and PC 36:6 (r = −0.220, *p* = 0.035, Figure 3B). The scores of PDQ-39 were negatively correlated with the levels of dihydro SM 24:0 (r = −0.281, *p* = 0.007), dihydro SM 22:0 (r = −0.353, *p* < 0.001), dihydro SM 20:0 (r = −0.328, *p* = 0.001) and PEp 38:6 (r = −0.300, *p* = 0.004), PC 38:5 (r = −0.261, *p* = 0.012) and PC 36:6 (r = −0.275, *p* = 0.008, Figure 3B). In the cognitive assessments, negative correlations were seen between the scores of NPI and levels of dihydro SM 24:0 (r = −0.236, *p* = 0.024), dihydro SM 22:0 (r = −0.264, *p* = 0.011) and dihydro SM 20:0 (r = −0.269, *p* = 0.010). The scores of MMSE were positively correlated with PEp 38:6 (r = 0.278, *p* = 0.007) and PC 38:5 (r = 0.247, *p* = 0.017). The levels of dihydro SM 24:0 (r = 0.239, *p* = 0.022), dihydro SM 22:0 (r = 0.211, *p* = 0.044), dihydro SM 20:0 (r = 0.245, *p* = 0.019) and PC 38:5 (r = 0.212, *p* = 0.043) were positively correlated with the scores of MoCA. The depression assessment showed negative correlations between the scores of BDI-II and the levels of dihydro SM 24:0 (r = −0.219, *p* = 0.036), dihydro SM 22:0 (r = −0.219, *p* = 0.036) and dihydro SM 20:0 (r = −0.243, *p* = 0.020). Positive correlations were observed between the scores of ADL and the levels of dihydro SM 24:0 (r = 0.241, *p* = 0.020), dihydro SM 22:0 (r = 0.282, *p* = 0.001) and dihydro SM 20:0 (r = 0.284, *p* = 0.006). The levels of ornithine demonstrated a positive correlation with those of α-synuclein (r = 0.221, *p* = 0.034). These linear correlations suggest lipid metabolites as potential biomarkers for disease evolution in the context of movement disability, cognitive dysfunction, and depression of PD.

### 3.4. Classification Model for Differentiating PD and HC

**Figure 4:**
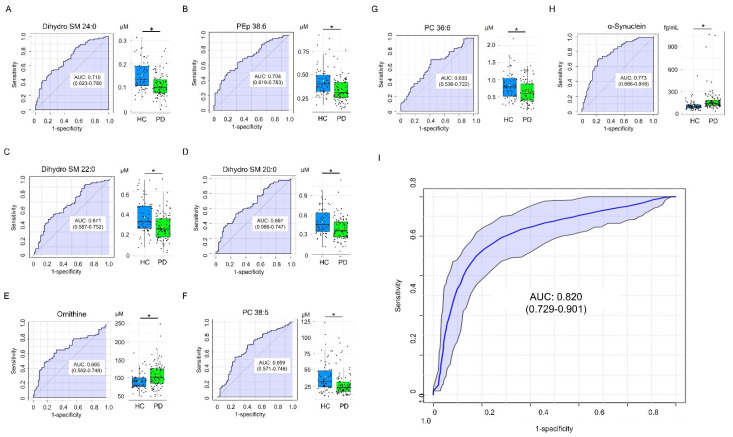
Diagnosis of PD using candidate metabolite markers. Receiver operating characteristic (ROC) curves and box plots for plasma levels of (**A**) dihydro sphingomyelin (SM) 24:0, (**B**) phosphatidylethanolamine-plasmalogen (PEp) 38:6, (**C**) dihydro SM 22:0, (**D**) dihydro SM 20:0, (**E**) ornithine, (**F**) phosphatidylcholine (PC) 38:5, (**G**) PC 36:6, and (**H**) α-synuclein for the diagnosis of Parkinson’s disease (PD). The area under the ROC curve (AUC) was in shadow. The black center line in box plots denoted the median, while the blue or green boxes contain the 25th to 75th percentiles. The black whiskers mark the 5th and 95th percentiles, and mean values were marked with yellow dots. *: Statistically significant between PD and the healthy controls (HC), *p* < 0.05, Two-tailed Student’s *t*-test. (**I**) ROC analysis on a combination of α-synuclein, dihydro SM 24:0, and PEp 38:6 by support vector machine. The 95% confidence band was in shadow.

ROC curve analysis was performed to evaluate the potential of selected metabolites as biomarkers for PD diagnosis (Figure 4). Dihydro SM C24:0 demonstrated greatest area under the ROC curve (AUC, 0.710, Figure 4A) to distinguish PD and HC, followed by PEp 38:6 (0.704, Figure 4B), dihydro SM 22:0 (0.671, Figure 4C), dihydro SM 20:0 (0.667, Figure 4D), ornithine (0.665, Figure 4E), PC 38:5 (0.659, Figure 4F) and PC 36:6 (0.633, Figure 4G). α-Synuclein also demonstrated good potential to differentiate PD from HC (AUC: 0.773, Figure 4H). The SVM algorithm using a combination of α-synuclein, dihydro SM 24:0, and PEp 38:6 demonstrated a better ability than α-Synuclein alone to separate PD from HC (AUC: 0.820, Figure 4I). These results support the potential of combining identified metabolite biomarkers with α-synuclein to establish a machine learning algorithm for PD diagnosis.

## 4. Discussion

**Figure 5:**
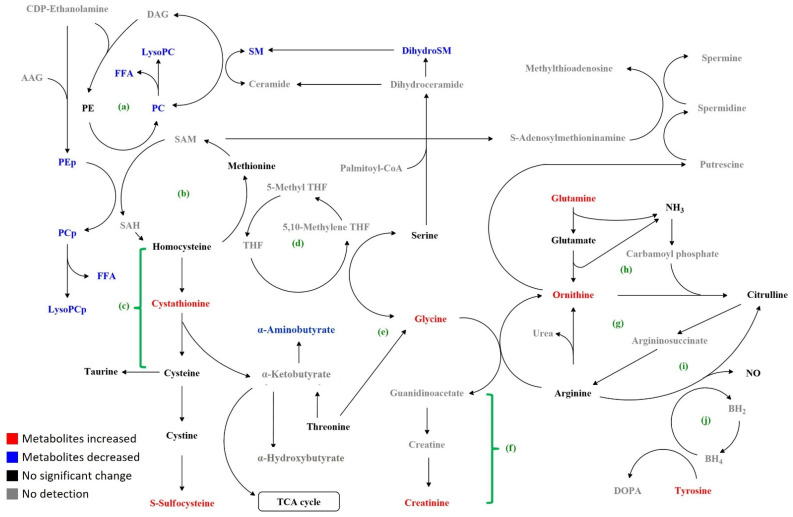
Map of metabolic changes in PD compared to the controls. (**a**) Sphingolipid and related glycerophospholipid biosynthetic pathways; (**b**) methionine cycle; (**c**) transsulfuration pathway; (**d**) folate cycle; (**e**) glycine, serine, and threonine pathway; (**f**) creatine synthesis pathway; (**g**) urea cycle; (**h**) ornithine–proline–glutamate pathway; (**i**) nitric oxide (NO) synthesis pathway; (**j**) biopterin cycle. AAG: alkyl acylglycerol; BH_4_: tetrahydrobiopterin; BH_2_: dihydrobiopterin; DAG: diacylglycerol; dihydroSM: dihydrosphingomyelin; DOPA: dopamine; FAA: free fatty acid; LysoPC: lysophosphatidylcholine; PC: phosphatidylcholine; PCp: phosphatidylcholine-plasmalogen; PE: phosphatidylethanolamine; PEp: phosphatidylethanolamine-plasmalogen; SM: sphingomyelin; SAM: S-S-adenosyl methionine; SAH: S-adenosyl homocysteine; TCA: tricarboxylic acid.

Previous studies have demonstrated a panel of metabolites with altered levels in PD patients (Table 3). By extensively examining the plasma levels of 255 metabolites in PD patients, we found profoundly metabolomic alterations in levels of 72 metabolites, involving sphingolipid and glycerophospholipid biosynthesis, transsulfuration, and metabolism of amino acids and amines (Table 2, Supplementary Table S1, and Figure 5). Among the identified 72 metabolites, only glycine has been found to be increased in PD patients previously (Bold in Table 3), which is similar to our results. 

We further selected 6 metabolites that demonstrated correlations with disease severity. Dihydro SM 24: 0, dihydro SM 22:0, dihydro SM 20:0, PC 38:5, and PEp 38:6 demonstrated the lowest level in PD patients at the advanced stage, followed by those at the early stage and HC. By contrast, ornithine showed the highest level in PD patients at the advanced stage, followed by those at the early stage and HC. The levels of these metabolites further showed linear correlations with the clinical scores for movement disability, cognitive dysfunction, and depression. The SVM machine learning algorithm using α-synuclein, dihydro SM 24:0, and PEp 38:6 demonstrated a good ability to differentiate PD from HC. In addition to the identification of a panel of candidate metabolic markers, these results also support the role of plasma metabolomic profiles in detecting PD and monitoring disease progression. 

SM contains acyl chains that vary in length from long-chain to very-long-chain fatty acids and is a kind of indispensable sphingolipid in mammalian cell membranes [28]. The levels of SM in the brains of PD patients were 42% reduced compared with the controls [29]. The cores of Lewy bodies also comprised large amounts of lipids, most importantly sphingomyelin and phosphatidylcholine [30]. SM is hydrolyzed by sphingomyelinase to produce phosphocholine and ceramide. The 1-methyl-4-phenyl-1,2,3,6-tetrahydropyridine (MPTP)-induced PD-like mice up-regulates sphingomyelinase expression and activity, resulting in a reduction in SM and an increase in ceramide [31]. Pharmacological inhibition of sphingomyelinase in Thy1-αSyn PD mouse model reduces α-synuclein aggregates in the substantia nigra and improves motor performance in a pole test [32]. Our study discovered the reductions of SMs in the plasma of PD patients, which is compatible with the finding in the substantia nigra of the PD mouse models. Importantly, the reduction of dihydro SM 24:0, 22:0, and 20:0 was particularly prominent in PD patients at the advanced stage. The linear correlation of levels of these dihydro SMs with UPDRS, PDQ-39, MoCA, NPI, BDI-II, HAM-D, and ADL further suggests the possible application of dihydro SMs as biomarkers indicative of PD progression in different clinical aspects. 

PC, which are essential components of cell membranes and lipoproteins, plays critical roles in membrane structure and cellular signaling [33]. Inhibition of PC synthesis can trigger apoptosis [34]. It has been shown that PCs were entangled in Lewy bodies [30]. Decreased levels of PC 34:5, 36:5, and 38:5 have been observed in the brains of PD patients [35]. Increased levels of PC 44:6 and 44:5 and decreased levels of PC 35:6 were reported in the plasma of PD patients [26]. In 6-hydroxydopamine-treated rats, most PCs in substantia nigra were also decreased [36]. PCs could also affect the conformation and aggregation of N-acetylated α-synuclein, which further enhances binding to micelles rich in PCs [37]. In addition to PD, low plasma phosphatidylcholine levels have been shown in patients with Alzheimer’s and Huntington’s disease [38,39]. Among 33 PCs and LPC showing reduced levels in PD patients, only PC 38:5 and PC 36:6 demonstrated significant differences between early and late disease stages. This specificity needs to be confirmed by further investigations. 

PE, the most abundant phospholipid in the brain [40], was synthesized by phosphatidylserine decarboxylase (PSD) (Figure 5). Inhibition of PSD in the *Caenorhabditis elegans* model of synucleinopathy enhanced the dopaminergic neuron degeneration, whereas supplementation with ethanolamine led to partial rescue [41]. Decreased levels of PEs were observed in the brains of early PD patients [42]. Our study found reduced plasma levels of 8 PEs in PD patients; of note, seven of them hada head group of plasmalogen (PEp). Decreased levels of PEps were also seen in the brains of patients with AD [43]. We further found the level of PEp 38:6 correlated with the scores of UPDRS, PDQ-39, and MMSE, indicating its potential as a marker for clinical deterioration. 

Glutamine, the most abundant free amino acid, is involved in mitochondria energy production, DNA damage response, apoptosis, and autophagy [44]. In the MPP^+^-treated PC12 cell model for PD, glutamine reduced cytotoxicity by suppressing the PI3K/Akt signaling pathway [45]. Derived from glutamine, ornithine is the precursor of major polyamines (putrescine, spermidine, and spermine), glutamate, and γ-aminobutyric acid. It is decarboxylated to putrescine by ornithine decarboxylase [46] (Figure 5). In a small case-control study, serum levels of ornithine was elevated in PD patients compared with the controls [47], however, larger prospective studies will be needed to clarify the role of glutamine and ornithine in PD. 

The transsulfuration pathway involves the transfer of sulfur from homocysteine to cysteine via cystathionine (Figure 5) [48,49]. Cysteine can be converted to other sulfur-containing molecules such as taurine and sulfocysteine. Elevated plasma cystathionine levels were associated with oxidative damage and endothelial dysfunction [49], and more pronounced in PD patients in disease progression [50]. Sulfocysteine is structurally highly similar to the excitatory neurotransmitter glutamate. The binding of sulfocysteine to N-methyl D-aspartate (NMDA) receptor leads to calcium influx, activates the protease calpain, and calpain-dependent degradation of the inhibitory synaptic protein gephyrin, and promotes loss of γ-aminobutyric acid-ergic synapses [51]. Our results showing increased cystathionine and sulfocysteine may implicate increased oxidative damage in PD patients. The amino group transfer reaction from cystathionine to α-ketobutyrate generates α-aminobutyrate, which exerts protective effects against oxidative stress [52] (Figure 5). Elevated levels of α-aminobutyric acid were observed in CSF of AD patients [53]. Derived from serine and threonine, glycine demonstrated an anti-oxidant effect against neurodegeneration [54]. Similar to our results, Iwasaki et al. found increased plasma levels of glycine in PD patients [27]. Elevation of extracellular glycine level by glycine transporter inhibitor promotes striatal axon sprouting in dopaminergic neurons [55]. These findings warrant further investigations to explore the potential of these amino acids as biomarkers and therapeutic targets for PD.

As a major constituent of Lewy bodies [1,56], α-synuclein is one of the two most studied molecules in PD. Although a number of studies have shown elevated plasma levels of α-synuclein levels in PD patients [57,58,59,60], inconsistent results were also reported [61,62,63,64]. No correlation was detected between α-synuclein level and disease stage in PD patients [57]. Although elevated α-synuclein level in PD patients was recapitulated by our study, its correlation with clinical parameters remained absent. However, our identified metabolites demonstrated significant correlations with scores for measuring movement disability, cognitive dysfunction, and depression, suggesting that the identified metabolites have greater potentials than α-synuclein as biomarkers to indicate disease severity. Furthermore, the diagnostic performance of α-synuclein in PD patients can be further improved by implementing SMV machine algorithm with α-synuclein, dihydro SM 24:0 and PEp 38:6, suggesting the potential of lipid metabolites as supplementary markers for PD diagnosis.

There are limitations to this study. The relatively higher proportion of PD patients at the early stage may conceal the alterations of metabolites in those at an advanced stage. The power may not be big enough to detect smaller changes of metabolites in PD. Some unknown interactions of medications or other factors may also contribute to the metabolic differences between groups. The results of patients of Taiwanese descent could be influenced by Asian diet and lifestyle. Nevertheless, our study clearly captures important features of metabolomics in the plasma of PD patients. These metabolic changes provide more potential avenues for investigating pathogenesis, monitoring clinical progression, and treatment efficacy in PD patients. It remains unclear whether the metabolites identified are specific to PD. In future, a comparison with serum or plasma from other neurodegenerative diseases, such as AD or amyotrophic lateral sclerosis, will produce important findings.

## Figures and Tables

**Table 1:** Demographic characteristics and blood biochemical parameters of included patients with Parkinson’s disease (PD) and the healthy controls (HC).

	HC	PD		
	(n = 60)	Early Stage (n = 71)	Advanced Stage (n = 21)	Total (n = 92)
Age (years)	67.35 ± 8.08	66.13 ± 10.61	72.71 ± 9.35	67.63 ± 10.65
Male (%)	30 (50.00)	37 (52.11)	11 (52.38)	48 (52.17)
Triglyceride (mg/dL)	103.35 ± 62.96	112.63 ± 64.34	90.90 ± 37.19	107.67 ± 59.77
Cholesterol (mg/dL)	184.05 ± 28.95	176.52 ± 37.06	170.67 ± 28.45	175.18 ± 35.22
Pre-prandial glucose (mg/dL)	99.12 ± 11.02	104.58 ± 20.19	97.71 ± 18.67	103.01 ± 19.95
BMI	24.41 ± 3.09	24.63 ± 3.53	23.63 ± 33.56	24.42 ± 3.56
UPDRS		28.49 ± 15.96	77.61 ± 36.35 ^d^	40.94 ± 29.17
UPDRS-part 3		17.50 ± 9.17	42.62 ± 15.13 ^d^	23.30 ± 15.15
Hoehn–Yahr stage		1.61 ± 0.48	3.02 ± 0.54 ^d^	1.93 ± 0.77
LEDD (mg)		471.95 ± 436.44	1323.74 ± 668.44 ^d^	668.38 ± 611.51
Diabetes (%)	3 (5.00)	11 (15.28) ^b^	1 (5.00) ^c^	12 (13.04) ^a^
CDR	0.20 ± 0.25	0.34 ± 0.24	0.64 ± 0.39 ^b,c^	0.41 ± 0.30 ^a^
MMSE	29.61 ± 8.98	27.32 ± 3.88	21.81 ± 6.43 ^b,c^	26.07 ± 5.11 ^a^
MoCA	27.95 ± 2.38	24.35 ± 5.73 ^b^	18.00 ± 8.41 ^b,c^	22.90 ± 6.93 ^a^
NPI	0.53 ± 1.70	2.06 ± 2.96 ^b^	7.57 ± 7.30 ^b,c^	3.32 ± 4.88 ^a^
BDI-II	1.67 ± 2.90	6.62 ± 5.08 ^b^	16.10 ± 7.11 ^b,c^	8.70 ± 6.81 ^a^
HAM-D	1.62 ± 2.73	5.30 ± 3.92 ^b^	11.40 ± 6.76 ^b,c^	6.64 ± 5.30 ^a^
ADL	99.92 ± 0.65	99.72 ± 1.44	69.05 ± 28.62 ^b,c^	92.72 ± 18.68 ^a^
PDQ-39	5.82 ± 8.26	22.42 ± 16.25 ^b^	67.33 ± 33.67 ^b,c^	32.67 ± 28.49 ^a^
α-Synuclein (fg/mL)	112.74 ± 70.25	182.85 ± 167.83 ^b^	209.25 ± 177.46 ^b^	188.43 ± 169.10 ^a^

ADL: activities of daily living; BDI-II: Beck Depression Inventory II; BMI: body mass index; CDR: Clinical Dementia Rating; HAM-D: Hamilton Depression Rating Scale; LEDD: Levodopa equivalent daily dose; MMSE: Mini-Mental State Examination; MoCA: Montreal Cognitive Assessment; NPI: Neuropsychiatric Inventory Questionnaire; PDQ-39: Parkinson’s Disease Questionnaire; UPDRS: Unified Parkinson’s Disease Rating Scale. ^a^: Statistically significant in comparison with HC (HC vs. total PD). *p* < 0.05. Two-tailed Student’s *t*-test. ^b^: Statistically significant in comparison with HC (HC vs. early stage PD vs. advanced stage PD). *p* < 0.05. One-way analysis of variance with Bonferroni correction. ^c^: Statistically significant in comparison with PD patients at the early stage. (HC vs. early stage PD vs. advanced stage PD). *p* < 0.05. One-way analysis of variance with Bonferroni correction. ^d^: Statistically significant in comparison with PD patients at the early stage (early stage PD vs. advanced stage PD). *p* < 0.05. Two-tailed Student’s *t*-test.

**Table 2:** Significantly changed levels of plasma metabolites in the patients with Parkinson’s disease (PD) compared to the healthy controls (HC).

Compound Name	HC (n = 60)	PD (n = 92)	*p* Value
SM 26:0	0.118 ± 0.026	0.095 ± 0.025	<0.001
Dihydro SM 24:0	0.152 ± 0.059	0.110 ± 0.048	0.0014
PEp 38:6	0.433 ± 0.158	0.332 ± 0.123	0.0014
5-Hydroxytryptophan	0.009 ± 0.002	0.015 ± 0.013	0.0015
PC 40:8	1.890 ± 0.530	1.509 ± 0.489	0.0015
SM 14:1	0.523 ± 0.150	0.418 ± 0.151	0.0016
FFA 18:0	21.333 ± 5.952	17.443 ± 4.427	0.0017
Glutamine	550.337 ± 75.570	602.440 ± 73.175	0.0018
PCe 38:6 and/or PCp 38:5	8.123 ± 2.597	6.406 ± 1.951	0.0019
PCe 36:4	21.490 ± 5.720	17.710 ± 4.745	0.0019
PEp 38:5	0.360 ± 0.136	0.275 ± 0.119	0.0028
FFA 20:0	0.387 ± 0.103	0.321 ± 0.100	0.0028
SM 16:1	15.502 ± 3.500	13.099 ± 3.900	0.0029
PC 34:0	5.756 ± 1.252	4.972 ± 1.218	0.0029
Ornithine	90.217 ± 22.245	107.580 ± 34.162	0.0030
PCe 36:5	2.448 ± 1.823	1.426 ± 1.122	0.0031
SM 23:0	12.424 ± 3.087	10.539 ± 2.787	0.0032
PC 28:1	2.412 ± 0.613	2.010 ± 0.658	0.0032
PCe 38:1	4.592 ± 1.166	3.875 ± 1.031	0.0032
PCe 36:1	1.911 ± 0.429	1.644 ± 0.438	0.0039

*p*-value: Two-tailed Student’s *t*-test with FDR correction. Concentration: μM. FFA: free fatty acid; PC: phosphatidylcholine; PCe: phosphatidylcholine-ether; PCp: phosphatidylchololine-plasmilogen; PEp: phosphatidylethanolamine-plasmalogen; SM: sphinogomyelin.

**Table 3:** Potential metabolite biomarkers for Parkinson’s disease in previous literature.

Candidate Marker	Origin	Change	Reference
Ethymalonate, myoinositol, propylene glycol, pyruvate, sorbitol	Plasma	↑ (PD versus HC)	[3]
Homovanillate, 3-methoxytyrosine, 3-methytyramine sulfate,	Serum	↑ (PD versus HC)	[4]
N1,N8-diacetylspermidine, N1,N12-diacetylspermine, N1-acetylspermidine, N1-acetylspermine, N8-acetylspermidine	Serum	↑ (PD versus HC)	[6]
Quinolinic acid	Plasma	↑ (PD versus HC)	[8]
Alanine, methionine, 2-oxoisocaproic acid, pyroglutamate, malate, serine	Plasma	↑ (PD versus HC)	[9]
8-Hydroxy-2-deoxyguanosin, glutathione	Plasma	↑ (PD versus HC)	[10]
L-arginyl-L-alanine, 1,3-dimethyluracil, Lyso-platelet activating factor C16, α-N-phenylacetyl-L-glutamine, PC 44:5, PC 44:6, sarcosine,	Plasma	↑ (PD versus HC)	[26]
Aspartate, glutamate and **glycine**	Plasma	↑ (PD versus HC)	[27]
Acetate, ascorbate, citrate, ethanolamine, galactitol, glucolate, gluconate, glutarate, glycerol, isocitrate, malate, methylamine, methylmalonate, suberate, succinate, threonate, trimethylamine	Plasma	↓ (PD versus HC)	[3]
Spermine	Serum	↓ (PD versus HC)	[6]
Hypoxanthine	Plasma	↓ (PD versus HC)	[7]
Kynurenic acid,	Plasma	↓ (PD versus HC)	[8]
Hexadecenoic acid, linoleic acid	Plasma	↓ (PD versus HC)	[9]
Uric acid	Plasma	↓ (PD versus HC)	[10]
Ethanolamine, L-glutamyl-L-isoleucine, N-lauroylglycine, PE 34:1, PC 35:6, SM d30:1, SM d32:1, SM d39:1	Plasma	↓ (PD versus HC)	[26]

↑: up-regulation; ↓: down-regulation; HC: healthy control; PC: phosphatidylcholine; PD: Parkinson’s disease. PE: phosphatidylethanolamine; SM: sphingomyelin. Bold indicates the metabolite consistently found in our study.

## Data Availability

The datasets generated during the current study are available from the corresponding author on reasonable request.

## Supplementary Materials

The following supporting information can be downloaded at: https://www.mdpi.com/article/10.3390/cells11030395/s1, Table S1: Significantly changed plasma metabolites in patients with Parkinson’s disease.
