# Supplementary material for: Alterations of Sphingolipid and Phospholipid Pathways and Ornithine Level in the Plasma as Biomarkers of Parkinson’s Disease
Source: Cells. 2022 Jan 24;11(3):395. doi: 10.3390/cells11030395 (PMC8834036; doi:10.3390/cells11030395)
Supplement: Supplementary file 1 [file cells-11-00395-s001.zip › cells-1552025-supplementary.pdf]

Supplementary Table S1. Significantly changed plasma metabolites in the patients with Parkinson's disease.

Compound name	Control (n = 60)	PD (n = 92)	*P* value
SM 26:0	0.118 ± 0.026	0.095 ± 0.025	< 0.001
Dihydro SM 24:0	0.152 ± 0.059	0.110 ± 0.048	0.0014
PEp 38:6	0.433 ± 0.158	0.332 ± 0.123	0.0014
5-hydroxytryptophan	0.009 ± 0.002	0.015 ± 0.013	0.0015
PC 40:8	1.890 ± 0.530	1.509 ± 0.489	0.0015
SM 14:1	0.523 ± 0.150	0.418 ± 0.151	0.0016
FFA 18:0	21.333 ± 5.952	17.443 ± 4.427	0.0017
Glutamine	550.337 ± 75.570	602.440 ± 73.175	0.0018
PCe 38:6	8.123 ± 2.597	6.406 ± 1.951	0.0019
PCe 36:4	21.490 ± 5.720	17.710 ± 4.745	0.0019
PEp 38:5	0.360 ± 0.136	0.275 ± 0.119	0.0028
FFA 20:0	0.387 ± 0.103	0.321 ± 0.100	0.0028
SM 16:1	15.502 ± 3.500	13.099 ± 3.900	0.0029
PC 34:0	5.756 ± 1.252	4.972 ± 1.218	0.0029
Ornithine	90.217 ± 22.245	107.580 ± 34.162	0.0030
PCe 36:5	2.448 ± 1.823	1.426 ± 1.122	0.0031
SM 23:0	12.424 ± 3.087	10.539 ± 2.787	0.0032
PC 28:1	2.412 ± 0.613	2.010 ± 0.658	0.0032
PCe 38:1	4.592 ± 1.166	3.875 ± 1.031	0.0032
PCe 36:1	1.911 ± 0.429	1.644 ± 0.438	0.0039
PEp 40:7	0.236 ± 0.094	0.183 ± 0.074	0.0040
SM 14:0	7.810 ± 2.002	6.573 ± 2.104	0.0042
Dihydro SM 22:0	0.539 ± 0.233	0.407 ± 0.187	0.0043
PCe 34:2	8.933 ± 3.136	7.143 ± 2.719	0.0047
SM 22:1	26.836 ± 6.141	23.298 ± 6.022	0.0064
Tyrosine	53.098 ± 11.068	61.335 ± 17.991	0.0064
PEp 40:6	0.298 ± 0.114	0.235 ± 0.099	0.0065
LPC 18:0	41.146 ± 10.272	35.311 ± 10.101	0.0066
Kynurenine	3.636 ± 0.630	4.245 ± 1.492	0.0066
PC 38:0	0.426 ± 0.110	0.365 ± 0.099	0.0068
Dihydro SM 20:0	0.327 ± 0.133	0.255 ± 0.119	0.0077
S-Sulfocysteine	1.955 ± 0.661	2.339 ± 0.725	0.0078
SM 21:0	4.736 ± 1.252	4.058 ± 1.180	0.0087
PEp 36:4	0.576 ± 0.231	0.457 ± 0.189	0.0089
PC 38:2a	9.404 ± 1.859	8.304 ± 2.227	0.0090
LPC(P-18:0)	0.105 ± 0.033	0.088 ± 0.030	0.0096
SM 13:0	0.126 ± 0.037	0.105 ± 0.044	0.0096
PC 38:6a	5.177 ± 1.601	4.360 ± 1.411	0.0106
SM 24:0	24.669 ± 6.613	21.267 ± 5.952	0.0108
SM 22:0	37.689 ± 9.289	32.830 ± 8.740	0.0108

PC 36:2	127.496 ± 30.656	111.396 ± 30.172	0.0114
PC 36:1b	17.233 ± 4.404	14.978 ± 4.102	0.0118
LPC(O-16:0)	1.607 ± 0.502	1.360 ± 0.424	0.0125
PC 40:4b	0.328 ± 0.111	0.271 ± 0.114	0.0167
Cystathionine	0.901 ± 0.306	1.237 ± 1.013	0.0204
PC 36:6	0.836 ± 0.397	0.654 ± 0.328	0.0211
Dihydro SM 14:0	0.214 ± 0.072	0.180 ± 0.067	0.0214
PC 40:6	69.239 ± 27.856	56.880 ± 21.313	0.0221
PCe 36:0	0.047 ± 0.012	0.041 ± 0.012	0.0226
LPC 22:0	0.055 ± 0.017	0.047 ± 0.018	0.0246
PC 38:5b	39.095 ± 23.968	28.602 ± 18.586	0.0250
Dihydro SM 16:0	7.713 ± 1.852	6.849 ± 1.817	0.0264
PC 36:4a	17.856 ± 5.902	15.168 ± 5.762	0.0311
PCp 36:4	16.665 ± 5.284	14.375 ± 4.524	0.0318
α-Aminobutyric acid	32.156 ± 7.427	28.802 ± 7.379	0.0331
PCe 36:3	0.583 ± 0.281	0.459 ± 0.258	0.0332
PC 38:4	72.429 ± 25.887	62.137 ± 17.599	0.0368
SM 23:1	9.847 ± 2.298	8.816 ± 2.397	0.0390
LPC 20:2	0.391 ± 0.132	0.336 ± 0.113	0.0409
PC 40:5	11.535 ± 3.377	10.046 ± 3.567	0.0415
Indoxylsulfate	5.217 ± 2.990	7.448 ± 7.273	0.0416
SM 15:0	6.125 ± 1.472	5.395 ± 1.969	0.0417
PCp 38:4	2.319 ± 0.827	1.987 ± 0.669	0.0418
PEp 38:4	0.740 ± 0.307	0.617 ± 0.249	0.0419
PCe 38:2	3.715 ± 0.899	3.326 ± 0.900	0.0422
PC 34:4	1.396 ± 0.719	1.121 ± 0.522	0.0435
SM 12:0	0.217 ± 0.104	0.175 ± 0.083	0.0435
Glycine	216.684 ± 50.821	240.564 ± 64.291	0.0435
FFA 16:1	5.832 ± 3.602	7.739 ± 5.584	0.0436
LPE 18:1	0.790 ± 0.369	0.952 ± 0.397	0.0436
PEp 36:5	0.094 ± 0.084	0.062 ± 0.056	0.0437
LPC(P-16:0)	0.782 ± 0.211	0.693 ± 0.212	0.0437

P value: Two-tailed Student's *t*-test with FDR correction. Concentration: μM.

FFA: free fatty acid; LPE: lysophosphatidylethanolamine (LPE); LPC: lysophosphatidylcholine; PC: phosphatidylcholine; PCe: phosphatidylcholine-ether; PCp: phosphatidylcholine-plasmalogen; PEp: phosphatidylethanolamine-plasmalogen; SM: sphingomyelin.
